# Computational recognition of LncRNA signatures in tumor-associated neutrophils could have implications for immunotherapy and prognostic outcome of non-small cell lung cancer

**DOI:** 10.3389/fgene.2022.1002699

**Published:** 2022-10-28

**Authors:** Zhuoran Tang, Qi Wang, Peixin Chen, Haoyue Guo, Jinpeng Shi, Yingying Pan, Chunyu Li, Caicun Zhou

**Affiliations:** ^1^ Tongji University Medical School Cancer Institute, Tongji University, Shanghai, China; ^2^ Department of Medical Oncology, Tongji University Affiliated Shanghai Pulmonary Hospital, Tongji University Medical School Cancer Institute, Tongji University, Shanghai, China; ^3^ Department of Integrated Traditional Chinese and Western Medicine, International Medical School, Tianjin Medical University, Tianjin, China

**Keywords:** non-small cell lung cancer, tumor-associated neutrophils, long noncoding RNA, immunotherapy, computational recognition

## Abstract

Cancer immune function and tumor microenvironment are governed by long noncoding RNAs (lncRNAs). Nevertheless, it has yet to be established whether lncRNAs play a role in tumor-associated neutrophils (TANs). Here, a computing framework based on machine learning was used to identify neutrophil-specific lncRNA with prognostic significance in squamous cell carcinoma and lung adenocarcinoma using univariate Cox regression to comprehensively analyze immune, lncRNA, and clinical characteristics. The risk score was determined using LASSO Cox regression analysis. Meanwhile, we named this risk score as “TANlncSig.” TANlncSig was able to distinguish between better and worse survival outcomes in various patient datasets independently of other clinical variables. Functional assessment of TANlncSig showed it is a marker of myeloid cell infiltration into tumor infiltration and myeloid cells directly or indirectly inhibit the anti-tumor immune response by secreting cytokines, expressing immunosuppressive receptors, and altering metabolic processes. Our findings highlighted the value of TANlncSig in TME as a marker of immune cell infiltration and showed the values of lncRNAs as indicators of immunotherapy.

## Introduction

Lung cancer is related with high mortality rates in China with non-small cell lung cancer (NSCLC) accounting for >80% of lung cancers (Zhu et al., 2017). The administration of immune checkpoint inhibitors (ICIs) in cancer therapy has had remarkable results (Yue et al., 2018; Dolladille et al., 2020; Galluzzi et al., 2020). For advanced non-small cell lung cancer (NSCLC), several clinical trials have confirmed that as first- or second-line treatment, ICIs are superior to platinum-based chemotherapy (Ko et al., 2018; Vansteenkiste et al., 2019; Chen et al., 2020). However, only 20%–40% of advanced NSCLC patients achieve sustained clinical benefits from PD-(L)1 inhibitor therapy, with most patients having primary or acquired resistance to immunotherapy (Socinski, 2014). Moreover, those who do not respond to immunotherapy may suffer immune-related adverse events (IRAE) and the high costs of anti-PD-(L)1 monoclonal antibody therapy (Khoja et al., 2017; Das and Johnson, 2019; Schoenfeld et al., 2019). Thus, effective biomarkers that distinguish potential responders from non-responders, and indicate patient clinical response in real-time are urgently needed to improve treatment outcomes.

The TME is comprised of a complex cell population that includes tissue-resident lymphocytes, fibroblasts, endothelial cells, and neurons that are present before tumorigenesis, as well as blood-derived cells recruited to tumor sites (Butturini et al., 2019). Immune cells are the main cellular components in tumors. Tumor-infiltrating myeloid cells, including tumor-associated macrophages (TAM), regulatory dendritic cells, tumor-associated neutrophils (TAN), myeloid-derived suppressor cells (MDSC), as well as tolerogenic dendritic cells (TOL-DC), facilitate the formation of immunosuppressive microenvironments (Schupp et al., 2019). These cells directly or indirectly inhibit the antitumor immune response by secreting cytokines, expressing immunosuppressive receptors, and altering metabolic processes, leading to tumor immune escape. Tumor-associated neutrophils (TANs) are a key part of tumor-infiltrating myeloid cells and are regularly detected in the TME. Clinically, TANs can be used to predict treatment outcomes and immunotherapy response (Nielsen et al., 2021). Transcriptomic studies have identified gene expression biomarkers as well as signatures for quantitative assessment of TANs, as well as for stratification based on prognoses and immunotherapeutic response (Lecot et al., 2019; Wu and Zhang, 2020).

Long non-coding RNA (lncRNAs) influence almost all biological processes and pathways, and their dysregulation is associated with various diseases. Additionally, lncRNAs have wide functional diversity due to their influence on gene expression levels at transcriptional, post-transcriptional and epigenetic levels (Rinn and Chang, 2012; Fatica and Bozzoni, 2014; Marchese et al., 2017; Bao et al., 2020). The correlation between lncRNAs and immune function has been reported. Recent studies have shown that lncRNAs are abundant with cell type specificity in various immune cell subsets (Rinn and Chang, 2012; Atianand et al., 2017; Chen et al., 2017; Zhou et al., 2017; Zhou et al., 2018). LncRNAs expression pattern has been correlated with infiltrations of immune cells into the TME (Hu et al., 2013; Ranzani et al., 2015; Sage et al., 2018; Wang et al., 2018; Zhao et al., 2021). Nevertheless, neutrophil-specific lncRNAs as well as their significance in assessing TANs and prediction of clinical outcomes and immunotherapeutic responses require further study.

Here, a computational framework is proposed for determining neutrophil-specific lncRNA expression levels and lncRNA signatures for TANs (TANLncSig) *via* integrative immune, lncRNA, and clinical profiling analyses. The TANLncSig’s ability to predict clinical outcome and response to immunotherapy by NSCLC patients was also investigated.

## Materials and methods

### Neutrophil-specific long noncoding RNAs screening

The data set can be obtained from the GEO database with series accession number GSE28490 (https://www.ncbi.nlm.nih.gov/geo/query/acc.cgi?acc=GSE28490), These included chip data on the expression of nine human immune cells (neutrophils, monocytes, B cells, eosinophils, CD4 T cells, NK cells, mDCs, CD8 T cells, and pDCs). The GEO2R tool from GEO was used for differential expression analysis. Using adjusted *p* = <0.05 and logFC >1 as cutoff thresholds identified 17 lncRNAs with high neutrophil-specific expression.

### Construction of risk scoring model

Clinical data and TCGA RNA-seq datasets for LUSC and LUAD were downloaded by the UCSC Xena browser (https://xenabrowser.net/). Lusc-LINC01272-neutrophils malignant/Luad-LINC01272-neutrophils malignant results from single cell sequencing datasets. First, a monovariate Cox regression analysis was used to find neutrophil-specific lncRNAs with prognostic value in LUSC and LUAD, and LASSO Cox regression was used to determine their risk scores. The multivariate Cox regression analysis (age, risk score, tumor stage, gender), Kaplan-Meier (KM) survival analysis and 3, 5, and 10 years survival AUCs were used to evaluate risk score.

### Correlation analysis between risk score and tumor clinical phenotype

Multivariate ANOVA was used to analyze differences between neutrophil-specific, highly expressed lncRNA and risk score in LUSC and LUAD samples at various TNM stages.

### Analysis of risk score related pathways

In LUSC and LUAD samples, genes with mean expression levels >1 were identified and their correlation with risk score analyzed. 1,000 genes with the highest absolute correlation coefficient value were selected from those with positive correlation coefficients (>0, *p* = <0.05) and those with negative correlation coefficients (<0, *p* = <0.05). ClusterProfiler for R was used to analyze GO terms of biological process (BP), Molecular function (MF), cellular component (CC), and KEGG pathway enrichment analyses. After gene enrichment, the adjusted *p*-value < 0.05 and the smallest TOP10 was selected for mapping.

### Development of tumor-associated neutrophils-derived long noncoding RNAs signature to judge the prognosis of immunotherapy for non-small cell lung cancer using machine learning

Pearson correlation analysis was used to determine correlations between risk score, neutropen-specific lncRNAs, and the expression of common immune checkpoint inhibitors and correlation heat maps drawn, with * denoting *p ≤* 0.01 while + denotes *p ≤* 0.05.

## Results

### Prognostic significance of neutrophil-specific long noncoding RNAs

**FIGURE 1:**
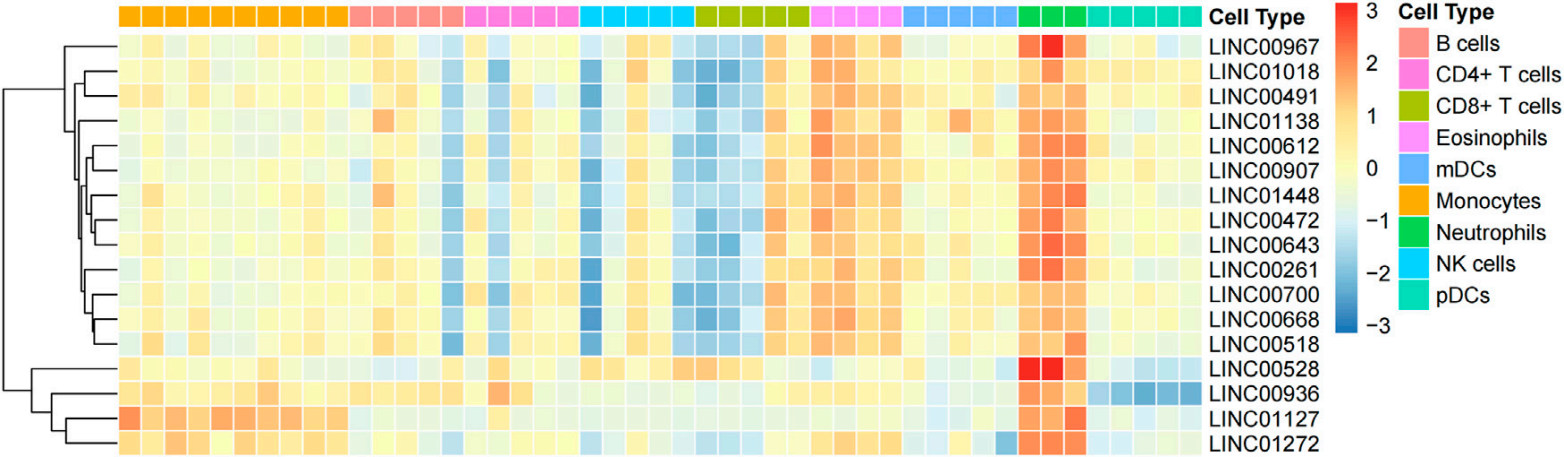
Using machine learning, 17 neutrophil-specific, highly expressed lncRNAs were identified. Differential expressions of lncRNAs between neutrophils and various immune cell types.

To recognize neutrophil-specific lncRNAs, dataset GSE28490 was downloaded from GEO (https://www.ncbi.nlm.nih.gov/geo/query/acc.cgi?acc=GSE28490). This dataset includes chip data on expressions of nine human immune cells (CD4^+^ T cells, neutrophils, monocytes, B cells, eosinophils, CD8^+^ T cells, NK cells, mDCs, and pDCs). Using GEO2R, 17 lncRNA specifically highly-expressed in neutrophils (*p* =<0.05, log2>1) were identified. These neutrophil-specific lncRNAs are referred to as TAN-associated lncRNAs (TANlncRNA) (Figure 1).

### Construction of a risk score based on neutrophil-specific long noncoding RNAs for prognosis prediction

**FIGURE 2:**
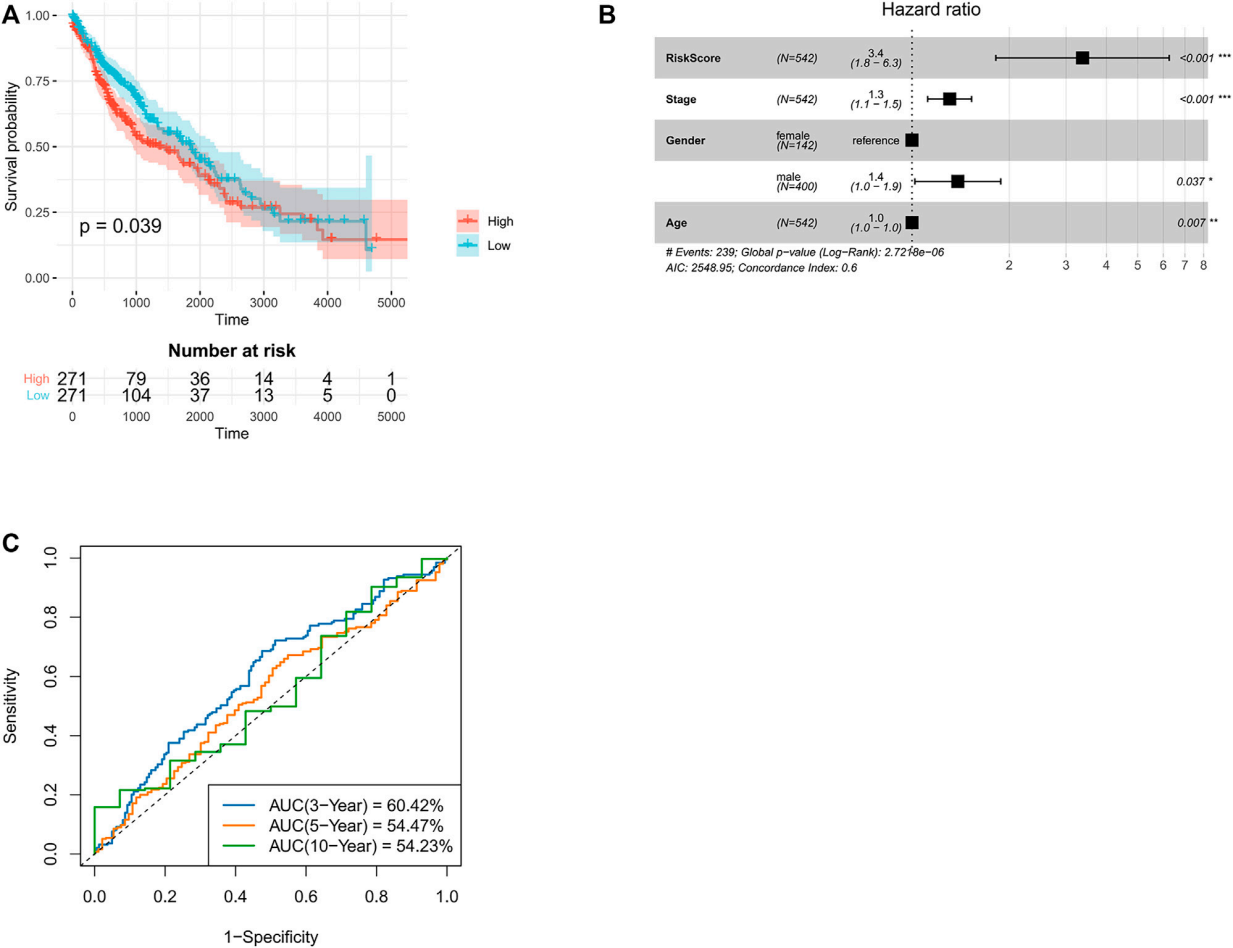
Validation of TANlncSig in TCGA lung squamous cell carcinoma discovery and testing datasets. **(A)** Kaplan-Meier survival analyses of lung squamous cell carcinoma patients. **(B)** Multivariate Cox regression analyses of patients with LUSC based on TCGA dataset. **(C)** ROC curve analyses of patients with LUSC using the TCGA dataset.

To develop a neutrophil-specific lncRNA risk score for predicting prognosis, the TCGA NA-SEQ dataset, TCGA lung squamous cell carcinoma (LUSC) as well as adenocarcinoma (LUAD) gene expression data, clinical features, and prognosis data were downloaded from UCSC Xena. First, univariate Cox regression analyses were used to establish neutrophil-specific lncRNAs with prognostic value in LUSC and LUAD. The final signature named TANlncSig (Table 1). This analysis identified three lncRNAs with prognostic value in LUSC (LINC01272, LINC00261, LINC00668, *p* = <0.05). Using these three lncRNAs, the expression value of lncRNA was weighted using multivariate Cox regression coefficient to obtain risk scores *via* the formula: risk score = 0.09 * LNC00668 + 0.17 * LNC00261. Then, TANlncSig scores for every patient in the discovery dataset were determined, after which the 542 patients were grouped into the high (*n* = 271) or low (*n* = 271) risk groups. Low risk group patients were found to have longer overall survival (OS) relative to the high-risk group patients (*p* = 0.039*, ≤*0.05, Figure 2A). Multivariate Cox regression analyses revealed that risk score (*p* < 0.001), stage (*p* < 0.001), age (*p* = 0.037, *≤*0.05), and gender (*p* = 0.007, *≤*0.01) significantly affected the prognostic outcomes of LUSC patients. The *p*-value and hazard ratio of TANlncSig were better than those of stage and age (Figure 2B). That said, TANlncSig has the potential to be a good predictor of efficacy. The predictive capacity of TANlncSig was authenticated using the TCGA internal testing dataset and revealed the 3-, 5-, and 10-year OS rates for low-risk group patients to be 60.42, 54.47, and 54.23%, respectively (Figure 2C). Indicating that risk score significantly correlates with OS in LUSC.

**TABLE 1:** Detailed information of six lncRNAs in the TANlncSig.

	Ensembl ID	Gene symbol	Location (GRCh37/hg19)	HR	Lower 0.95	Upper 0.95	*p*-value
LUAD	ENSG00000259974	LINC00261	*chr20:22,541,191–22,559,280*	0.8726407	0.7771	0.98	0.021359
	ENSG00000269220	LINC00528	*chr22:18,260,056–18,262,247*	0.5049413	0.2662	0.9577	0.036422
	ENSG00000253138	LINC00967	*chr8:67,104,349–67,109,554*	0.0026941	9.72E-06	0.7471	0.039252
LUSC	ENSG00000259974	LINC00261	*chr20:22,541,191–22,559,280*	1.2676382	1.1	1.461	0.001087
	ENSG00000265933	LINC00668	*chr18:6,925,473–6,929,868*	0.8529687	0.7512	0.9685	0.01412
	ENSG00000224397	LINC01272	*chr20:48,884,015–48,896,333*	1.14005	1.022	1.272	0.018818

**FIGURE 3:**
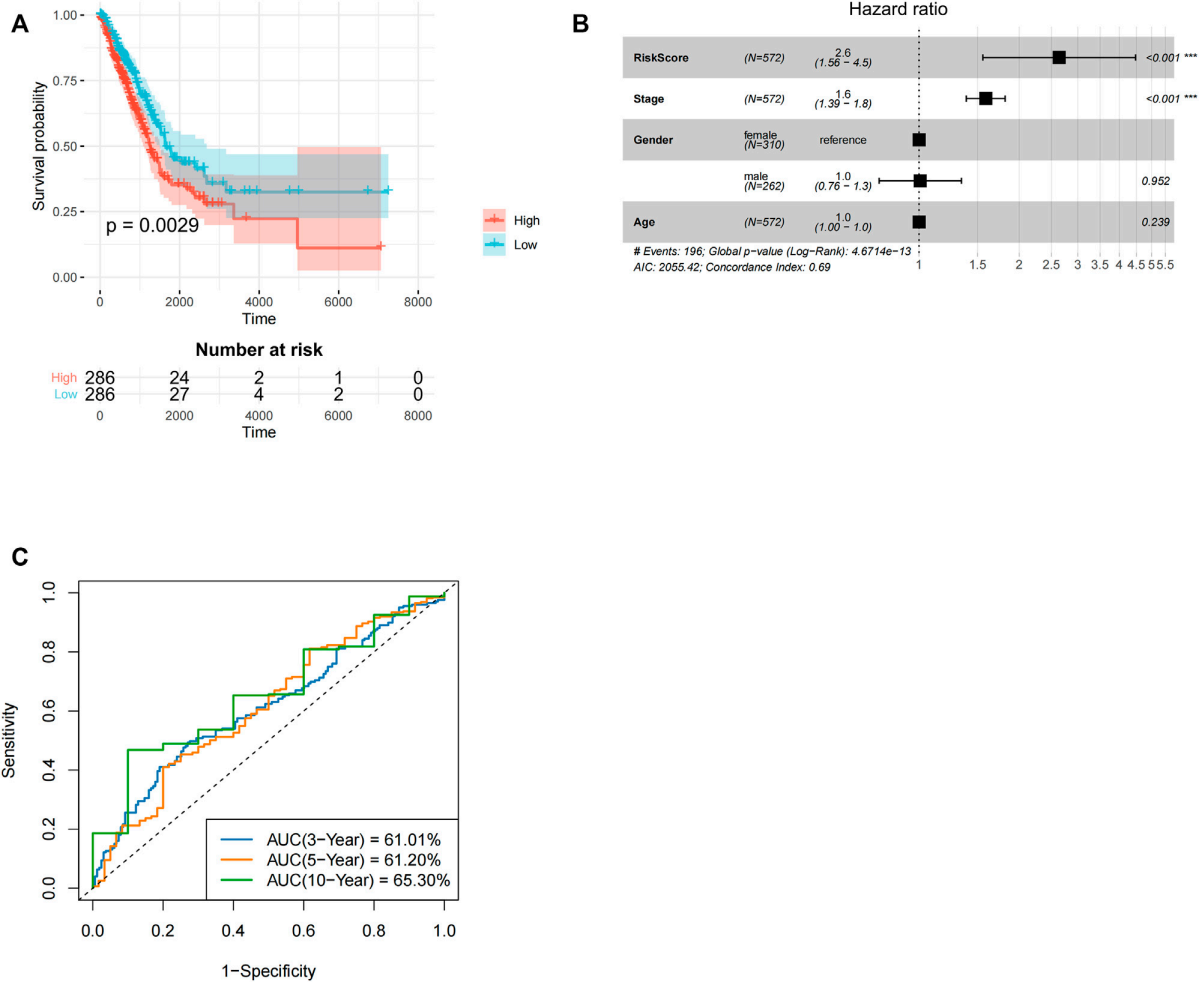
Development and subsequent validation of TANlncSig in the lung adenocarcinoma TCGA testing as well as discovery datasets. **(A)** Kaplan-Meier survival analyses for LUAD patients. **(B)** An analysis of the TCGA dataset of LUAD patients using multivariate Cox regression. **(C)** ROC curve analyses of patients with LUAD in the TCGA dataset.

Similar analysis was done for LUAD. First, three lncRNAs with prognostic values (LINC00528, LINC00967, and LINC00261) were identified using univariate Cox analysis. Using the above three lncRNAs, lncRNAs expression value was weighted by multivariate Cox regression coefficient to determine risk score using the formula: risk score = −5.32 * LINC00967-0.16 * LINC00261-0.74 * LINC00528. Patients with LUAD in the low-risk group had longer OS relative to high-risk group LUAD patients (*p* = 0.0029*, ≤*0.01, Figure 3A). Cox multivariate regression analyses revealed that risk score (*p* < 0.001) and stage (*p* < 0.001) significantly correlated with LUAD prognosis. In lung adenocarcinoma, the *p*-value and hazard ratio of TANlncSig were equally better than those of stage and age (Figure 3B). The 3-, 5-, and 10-year OS rates in low-risk group patients were 61.01, 61.20, and 65.30%, respectively (Figure 3C). These results indicate that risk scores in the LUAD dataset significantly correlate with patients’ OS.

### Correlation analysis between risk score and tumor clinical phenotype

**FIGURE 4:**
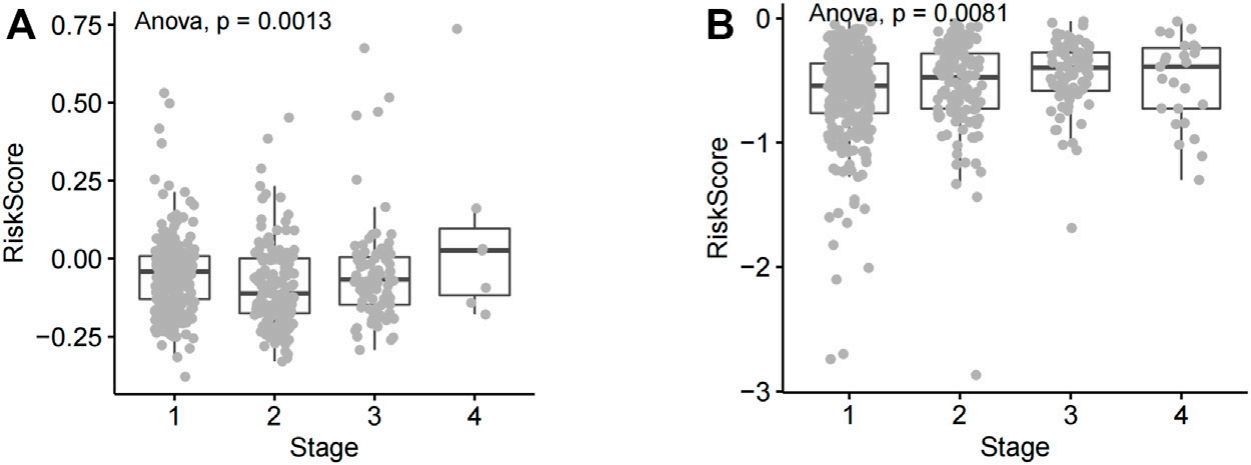
Analysis of risk score differences across NSCLC tumor stages. **(A)** The risk score of lung squamous cell carcinoma patients at various disease stages. **(B)** Risk scores of different lung adenocarcinoma stages.

Clinical phenotypic correlation analysis of single prognostic lncRNA and risk score (tumor stage, T, N, and M staging) was performed in lung adenocarcinoma as well as squamous cell carcinoma. According to statistical analysis, the risk score in different tumor stages of lung squamous cell carcinoma showed significant statistical differences, and the statistical results showed that *p* = 0.0013, <0.01 (Figure 4A). The risk score in different tumor stages of lung adenocarcinoma also showed significant statistical differences (*p* = 0.0081, <0.01) (Figure 4B).

**FIGURE 5:**
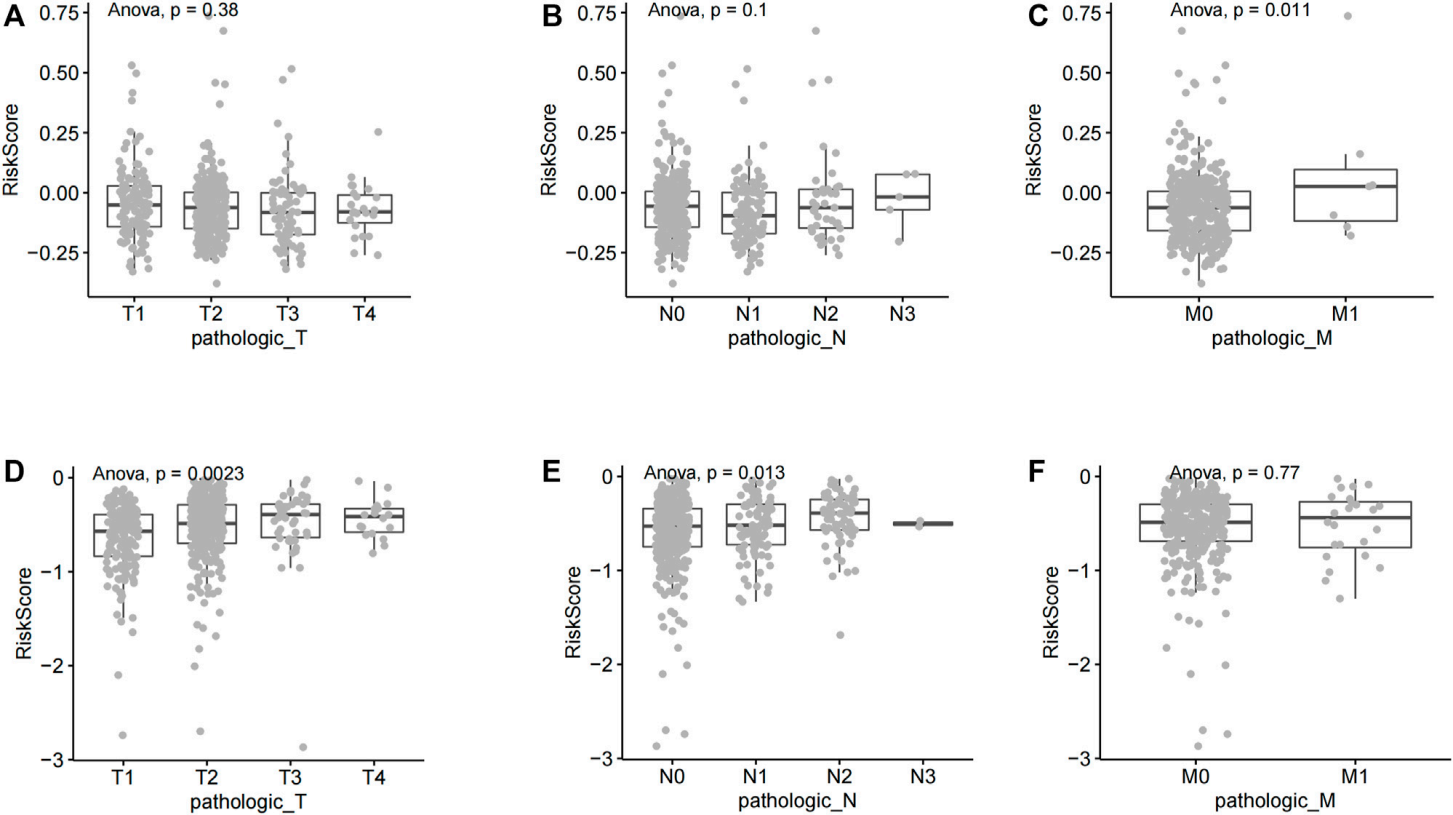
Correlation between riskscore and different TNM stages of non-small cell lung cancer. **(A)** A comparative analysis of risk scores in LUSC T staging. **(B)** A comparative analysis of risk scores in LUSC N staging. **(C)** A comparative analysis of risk scores in LUSC M staging. **(D)** A comparative analysis of risk scores in LUAD T staging **(E)** A comparative analysis of risk scores in LUAD N staging. **(F)** A comparative analysis of risk scores in LUAD M staging.

The TNM staging system is the most widely used tumor staging system, worldwide. T denotes tumor sizes and local invasion range, N denotes lymph node involvement, and M denotes distant metastasis. TNM staging has great clinical value in prognosis prediction (Ficarra et al., 2007; Moch et al., 2009). The risk score lack of significance in different T stages and N stages of lung squamous cell carcinoma (Figures 5A,B). The risk score has significant statistical difference in different M stages of lung squamous cell carcinoma (*p* = 0.011, <0.05) (Figure 5C). The risk score has significant statistical difference in different T stages (T1, T2, T3, and T4 stages) of lung adenocarcinoma (*p* = 0.0023, <0.01) (Figure 5D). Similarly, the risk score has significant statistical difference in different N stages (N0, N1, N2, and N3 stages) of lung adenocarcinoma (*p* = 0.013, <0.05) (Figure 5E). The risk score lack of significance in different M stages of lung adenocarcinoma (Figure 5F).

### Riskscore correlation pathway analysis

**FIGURE 6:**
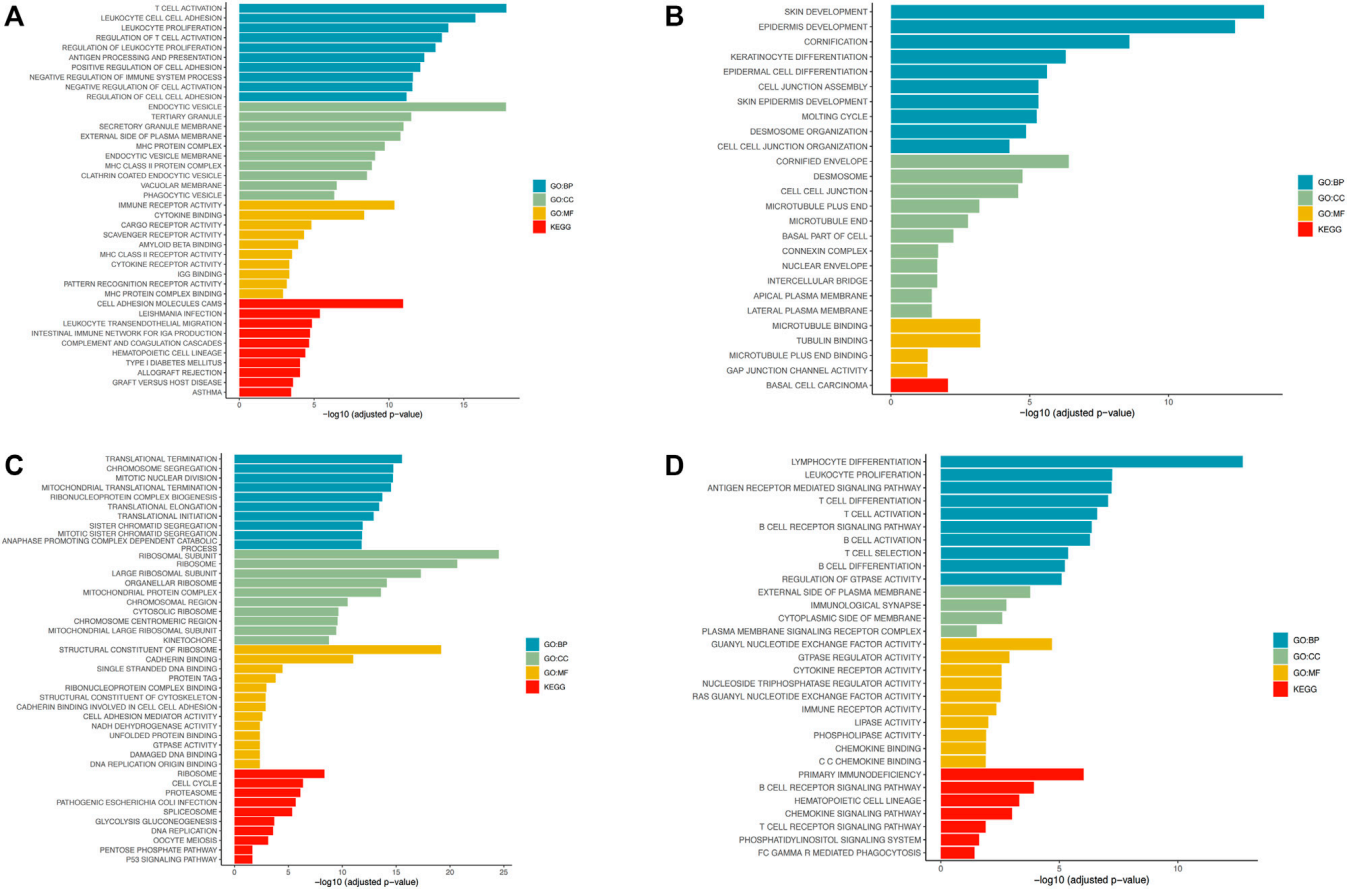
Pathway enrichment analysis using R’s cluster profiler. **(A)** Enrichment of positive correlation gene pathways in lung squamous cell carcinoma. **(B)** Enrichment of negative correlation gene pathways in lung squamous cell carcinoma. **(C)** Enrichment of positively correlation gene pathways in lung adenocarcinoma. **(D)** Enrichment of negative correlation gene pathways in lung adenocarcinoma.

In LUSC and LUAD samples, genes with average expression levels >1 were identified and their risk scores analyzed. 1,000 genes with the largest absolute correlation coefficient values were selected from positive (correlation coefficient >0, *p ≤* 0.05) and negative (correlation coefficient <0, *p ≤* 0.05) and correlation genes and pathway enrichment analysis done using cluster profiler on R. In LUSC, positive correlation genes are mainly associated with biological processes (BP) associated with T-cell activation, leukocyte proliferation, and leukocyte cell-cell adhesion. For cellular component (CC) they were enriched in endocytic vesicle, tertiary granule, and secretory granule membrane. For molecular function (MF), they were enriched in immune receptor activity and cytokine binding. KEGG pathway analysis revealed enrichment mainly for cell adhesion molecules cams (Figure 6A). Negative correlation genes in lung squamous cell carcinoma are mainly enriched for biological processes (BP) associated with skin development, epidermis development, and cornification. For cellular component (CC), they were enriched for cornified envelope, desmosome, and cell-cell junction. For molecular function (MF), they were enriched for microtubule binding and tubulin binding. For KEGG pathways, they were enriched for basal cell carcinoma (Figure 6B). Positive correlation genes in lung adenocarcinoma were mainly enriched in biological processes (BP) associated with translational termination and adenocarcinoma. For cellular component (CC), they were enriched for ribosomal subunits, ribosome and large ribosomal subunit. For molecular function (MF) they were enriched for structural constituent of ribosome and cadherin binding. For KEGG pathways, they were enriched for ribosome and cell cycle (Figure 6C). Genes associated with negative correlations in LUAD are involved in biological processes (BPs) associated with lymphocyte differentiation, leukocyte proliferation, and antigen receptor-mediated signaling. For cellular component (CC), they were enriched for external side of plasma membrane and immunological synapse. For molecular functions (MFs), they were enriched for guanyl-nucleotide exchange factor activity. For KEGG pathways, they were enriched for primary immunodeficiency and B-cell receptor signaling pathway (Figure 6D).

### The TANlncSig associates with tumor-associated neutrophils

**FIGURE 7:**
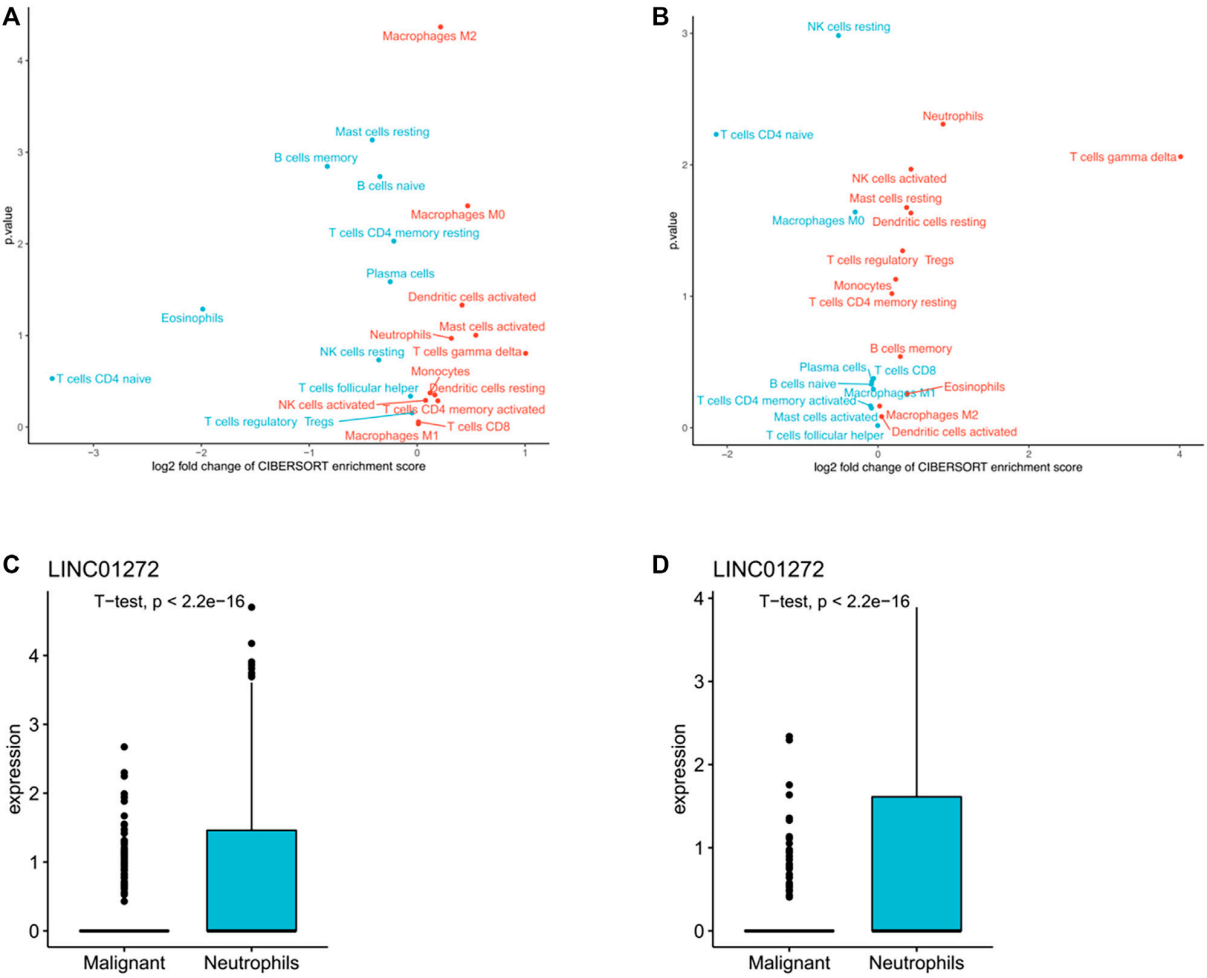
**(A,B)** An analysis of tumors with high and low TANlncSig based on the NES score from the GSEA in LUSC and LUAD provided the volcano plots for the enrichment of immune cell subpopulations. **(C,D)** The boxplots show lncRNA expression in both LUSC and LUAD neutrophil cell lines.

In accordance with previously reported expression levels of the immune cell specific marker genes, cibersort (https://cibersort.stanford.edu/) was further used to evaluate the levels of immune infiltration of 22 immune subpopulations in high-risk and low-risk patient groups. *t*-test was performed to determine the difference in lymphocyte infiltration levels between the two groups. As shown in Figures 7A,B, in both LUSC and LUAD, high-risk patients were significantly enriched in 12 immune subpopulations, while low-risk patients were enriched in 10 immune subpopulations. Additionally, mononuclear immune cells, including neutrophils, were found to infiltrate significantly more in the high-risk patient group than in several other groups. Single-cell sequencing data of LUSC and LUAD downloaded from GSE127465, cell type notes downloaded from TISCH (http://tisch.comp-genomics.org/). The homologous expression levels of LINC01272 of the TANlncSig in neutrophil cell lines differed significantly from those of malignant cell lines according to a subsequent analysis of neutrophil cell lines (Figures 7C,D). This indicates that these lncRNAs are expressed differently in neutrophils compared with malignant cells. In the above study, we found that the TANlncSig was not only associated with patient prognosis but also as a TAN indicator.

### TANlncSig was validated over several independent datasets using a microarray platform for prognostic value

**FIGURE 8:**
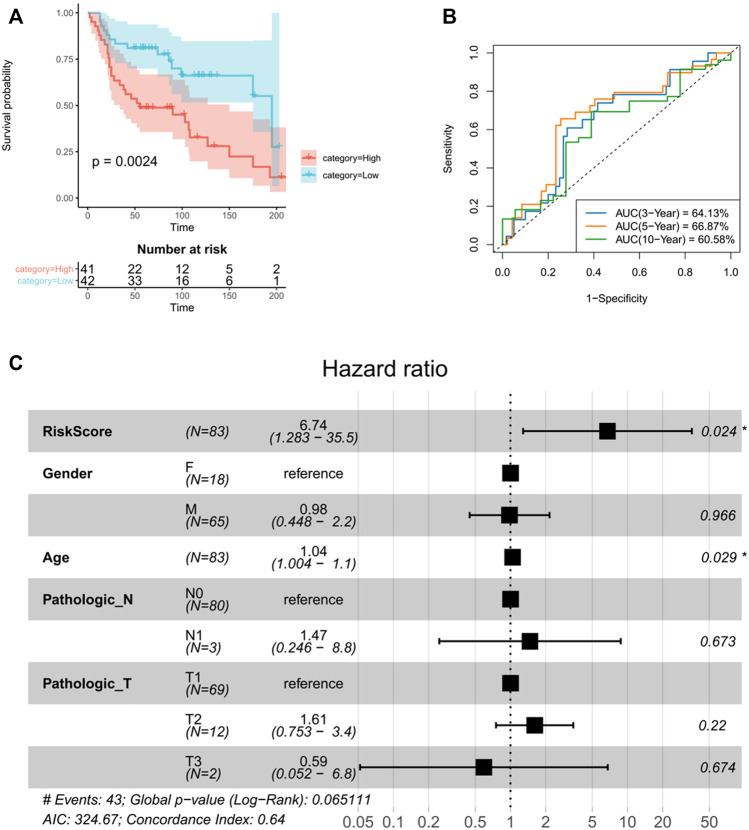
The TANlncSig was independently validated in the GSE30219 dataset **(A)**. Kaplan–Meier survival curves of OS were plotted between high- and low-risk groups stratified by the TANlncSig. **(B)**Time ROC curve of luad patients the GSE30219 dataset. **(C)** Visualization of the HRs from a multivariate Cox analysis of the TANlncSig and clinicopathological factors in GSE30219.

TANlncSig was further validated in independent datasets by the microarray platform in order to verify versatility and robustness of TANlncSig. The Affymetrix HG-U133 Plus 2.0 platform was used to analyze 83 LUAD patients from the GSE30219 dataset. As demonstrated again, TANlncSig can distinguish between patients who have high and low survival risk. A total of 83 patients were stratified into 41 high-risk patients and 42 low-risk patients in the GSE30219 dataset. Furthermore, patients in the high-risk group had a marginally poorer outcome than those in the low-risk group (*p* = 0.0024, *≤*0.01; log-rank test) (Figure 8A). The AUC of ROC curve at 3, 5, and 10 years were 64.13, 66.87, and 60.58% respectively (Figure 8B). The results show that TANlncsig can accurately predict the 5-year overall survival of patients, indicating that TANlncsig has good efficacy and certain stability. In order to investigate whether TANlncSig is an independent prognostic factor, a multivariate Cox regression analysis was conducted in patient cohorts. In the independent GSE30219 dataset, the TANlncSig still maintained a significant correlation with OS in the multivariate analysis (HR = 6.74, 95% CI 1.283-35.5, *p* = 0.024, *≤*0.01). Thus, these results demonstrate that the TANlncSig helps predict OS independently of other conventional clinical factors (Figure 8C).

### Significance of TANlncSig as a marker of immunotherapy

**FIGURE 9:**
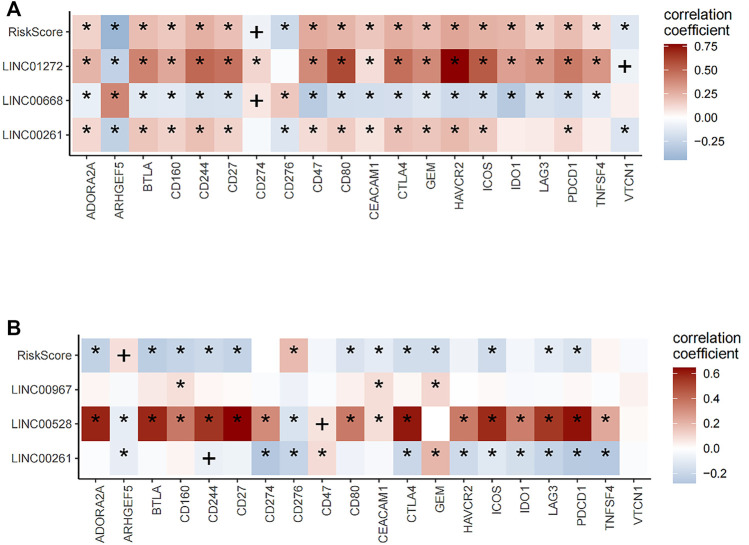
Correlation analysis of lncRNA and riskScore with expression levels of immune checkpoint blockade (ICB). **(A)** In lung squamous cell carcinoma, risk score, LINC01272, and LINC00261 positively correlated with the expression of most ICBs, while LINC00668 had negative correlations with the expression of most ICBs. **(B)** In lung adenocarcinoma, risk score had a negative correlation with the expression of most ICBs, while LINC00528 positively correlated with the expression of most ICBs.

Next, prognostic lncRNAs and risk score were correlated with immune checkpoint molecules expression in LUSC and LUAD patients. In LUSC, risk score, LINC01272, and LINC00261 positively correlated with the expression of most ICBs, while LINC00668 had negative correlations with the expression of most ICBs (Figure 9A). In LUAD, risk score had negative correlations with expression levels of most ICBs, while LINC00528 positively correlated with expression levels of most ICBs (Figure 9B). The expressions of risk score were divided into high and low groups and combined according to the median. The combination was used to analyze the prognosis of immunotherapy for non-small cell lung cancer. In lung squamous cell carcinoma, the combination of CEACAM1, TNFSF4, gem, CD47, vtcn1 and risk score can well stratify the prognosis of patients. In lung adenocarcinoma, all ICB molecules combined with risk score can well predict the prognosis of patients. These results suggest that risk score can be used as an index to predict the response of patients to immunotherapy.

## Discussion

In the peripheral blood, neutrophils are the most abundant white blood cells (Dinh et al., 2020). They have a central role in human non-specific immunity. Previous studies suggest that neutrophils inhibit tumors by secreting cytokines and producing reactive oxygen species (Vaughan and Walsh, 2005; Mishalian et al., 2013; Coffelt et al., 2015; Ponzetta et al., 2019). However, other studies indicate that neutrophils in the tumor microenvironment (TME) promote tumorigenesis. Cytokines and chemokines production by invasive neutrophils might affect the recruitment and activation of inflammatory cells in the TME, create an immunosuppressive microenvironment that is conducive for tumorigenesis, regulate tumor growth, metastasis and angiogenesis, and influence patient prognosis. Traditional methods for quantifying tumor immune cells infiltration based on histology or immunohistochemistry may have bias and variabilities (Yoshihara et al., 2013; Gibney et al., 2016; Spranger and Gajewski, 2018; Zhang et al., 2020; Sanchez-Pino et al., 2021). More recently, RNA-seq analyses have shown that lncRNAs exhibit a better degree of cell type specificity, relative to protein-coding genes in immune cells, highlighting their potential as subpopulation-specific immune cells molecular markers (Huang et al., 2018; Chen et al., 2019; Zhou et al., 2021).

Here, we used a machine learning-based computational framework to identify lncRNA features for evaluating TANs and explored their clinical significance using a combination of lncRNA, immune, and clinical spectrum analyses. The computational framework was used on the TCGA discovery dataset of NSCLC to identify a lncRNA signature (TANlncSig) comprised of 17 lncRNAs obtained from a list of neutrophil-specific lncRNAs using machine learning. Functional enrichment analysis of TANlncSig-related mRNAs showed that TANlncSig is highly correlated with cancer markers of immune response and sustained proliferative signals. Recent experimental evidence on some TANlncSig components is consistent with functional annotations using bioinformatics. It appears that Mir-1303, which is upregulated in tumor tissues, acts as a sponge for LINC01272 and negatively correlates with its expression. A reduction in LINC01272 expression in tissues and cells of NSCLC patients may serve as an independent prognostic marker. LINC01272 overexpression may inhibit NSCLC cells proliferation, migration, and invasion by inhibiting MI-1303 (Zhang and Zhou, 2021). LINC00261 downregulation in gastric cancer is associated with poor prognosis. Ectopic LINC00261 expression disrupts cell migration and invasion, inhibiting metastasis *in vitro* as well as *in vivo*. LINC00261 downregulation promotes cell migration and invasion *in vitro*. LINC00261 overexpression influences epithelial-mesenchymal transition (EMT) through the regulation of E-cadherin, Vimentin and N-cadherin (Liu et al., 2020; Zhai et al., 2021). LINC00668 expression is significantly upregulated *via* STAT3 signaling in NSCLC tissues as well as cell lines. Clinical studies show that upregulated LINC00668 correlates with histological grade, advanced TNM stage, and lymph node metastasis. Additionally, multivariate analyses established that LINC00668 as an independent marker of overall survival (OS) in patients with NSCLC. LINC00668 downregulation inhibits proliferation, migration, and invasion of NSCLC cells and promotes apoptosis. Mechanistically, LINC00668 is a direct target of miR-193a, leading to down-regulation in the expression of its target gene KLF7. STAT3-initiated LINC00668 promotes NSCLC progression by upregulating KLF7 *via* sponging Mir-193a. Therefore, it may serve as a prognostic marker and therapeutic target for NSCLC (An et al., 2019). From the perspective of lncRNA, TANlncSig seems to be a transcriptional marker as a potentially measurable indicator of neutrophil activity and prognosis.

To further assess TANlncSig’s role in clinical risk stratification, we evaluated its relationship with survival in patients with NSCLC. When applied to the TCGA RNAseq patient dataset, TANlncSig significantly correlated with patient survival. In TANlncSig, three lung squamous cell carcinoma, neutrophil-specific lncRNAs (LINC01272, LINC00261, and LINC00668) were markedly associated with prognostic outcomes. In lung adenocarcinoma, three neutrophil-specific lncRNAs (LINC00528, LINC00967, and LINC00261) significantly correlated with prognosis. In squamous cell carcinoma and lung adenocarcinoma, correlation analysis of individual lncRNAs and risk score with clinical features (TNM staging) revealed that risk score varied significantly with tumor stage. After adjusting for traditional clinical factors, TANlncSig was verified to be an independent prognostic marker for differentiating between poor and good survival outcomes across patient datasets.

Immune checkpoint inhibitors (ICIs) have emerged as effective lung cancer immunotherapies (Suresh et al., 2018; Iams et al., 2020). Some of the drugs acting on the immune checkpoints, CTLA4 and PD-1/PD-L1, have excellent performance against various tumors. Although significant breakthroughs have been made on CTLA4 and PD-1/PD-L1 antibodies, single-drug effective rates are only about 20%, and they benefit a limited proportion of patients (Magiera-Mularz et al., 2017; Lingel and Brunner-Weinzierl, 2019; Rotte, 2019; Yang and Hu, 2019; Liu and Zheng, 2020). The limited efficacy is attributable to the immune system’s complexity. Indeed, immune cells, cytokines, and immune adjuvants in the TME interact with each other, limiting the effects of drugs on individual targets. Thus, drugs that target different links and aspects of tumor immunity are needed to enhance immunotherapy outcomes. Up to 29 immunoglobulin superfamily members and 26 members of the tumor necrosis factor receptor superfamily are expressed on T-cell surfaces alone, and there have been preclinical or clinical studies on related immune targets and drugs. Specific immune checkpoints include lymphocyte activating gene 3 (LAG-3), T-cell immunoglobulin mucin 3 (TIM-3), and V region Ig inhibitor (VISTA). Non-specific immune checkpoints include human killer cell immunoglobulin like receptor (KIR), indoleamine 2, 3-dioxidase (IDO), and CD47, these novel immune checkpoint molecules are expected to provide hints for clinical and basic research (Manser et al., 2015; Munn and Mellor, 2016; Burugu et al., 2018; Huang et al., 2020; Logtenberg et al., 2020). VISTA, (B7-H5, PD-1H) is an immunomodulatory receptor that inhibits T-cell response. VISTA is overexpressed on CD11b myeloid cells (e.g., macrophages, monocytes, neutrophils, and dendritic cells) and it is found that in humans and mice at a lower level in primitive CD4^+^ and CD8^+^ T-cells as well as Tregs. With two potential protein kinase C binding sites and proline residues acting as docking sites in its cytoplasmic tail domain, VISTA can serve as both a receptor and a ligand (Huang et al., 2020; Mutsaers et al., 2021). OX40 (TNFRSF4) has been found to be expressed in activated NK cells, T-cells, NKT cells, as well as neutrophils, and acts as an auxiliary costimulatory immune checkpoint (Curti et al., 2013; Aspeslagh et al., 2016; Buchan et al., 2018). Combining immune checkpoint genes and TANlncSig showed combined prognostic effects on patient survival, in line with previous findings that immunomotor interactions between neutrophilic infiltration and expression levels of checkpoint genes affect the outcome of cancer patients and immunotherapy may also be associated with this condition. In combination with earlier findings, it appears that TANlncSig is correlated with immunosuppressive phenotypes and could predict ICI response. Together, these results indicate that TANlncSig can complement and/or add information to existing immune checkpoint genetic markers.

Due to few gene mutations, lung squamous cell carcinoma is less selective than adenocarcinoma with regards to treatment options, and its survival time (about 1 year) is shorter than that of adenocarcinoma (Travis et al., 2021). Thus, novel, effective advanced lung squamous cell carcinoma treatments are needed to improve patient outcomes. The emergence of immune checkpoint inhibitors in recent years has markedly improved treatment options for advanced lung squamous cell carcinoma patients. Immune checkpoint inhibitors have substantially changed advanced lung squamous cell carcinoma treatment, leading to a shift from retro line immunotherapy to front-line treatment options. Originally approved as second-line treatment after platinum-based dual therapy, palivizumab is now recommended as a single-agent first-line treatment or in combination with chemotherapy. Although treatments targeting the immune checkpoints PD-1 and CTLA4 are successful in many cancers, not all patients benefit from them. Our findings indicate that the combination of CEACAM1, TNFSF4, GEM, CD47, VTCN1, and TANlncSig in squamous cell carcinoma can effectively stratify patients by prognosis, highlighting these immune checkpoint receptors as potential therapeutic targets against advanced lung cancer.

## Conclusion

In conclusion, we used a machine learning-based computational framework to identify lncRNA features of TANs (TANlncSig) *via* comprehensive analyses of lncRNA, immune, as well as clinical features. TANlncSig revealed a substantial and repeatable correlation with outcomes, even after adjustments of clinical covariates. Analysis of correlation between prognostic lncRNAs and risk score with the expression of immune checkpoint molecules demonstrated that TANlncSig can predict immunotherapy. The study is the first to define lncRNA characteristics of tumor-associated neutrophils, highlighting the importance of lncRNAs in immune responses and the potential for more precise and personalized treatment cancer immunotherapy.

## Data Availability

The original contributions presented in the study are included in the article/supplementary material, further inquiries can be directed to the corresponding authors.
